# Investigation of the relationships between the alveograph parameters

**DOI:** 10.1038/s41598-021-84959-3

**Published:** 2021-03-05

**Authors:** Anne-Sophie Schou Jødal, Kim Lambertsen Larsen

**Affiliations:** 1 Department of Chemistry and Bioscience, Section of Chemistry, Aalborg University, Fredrik Bajers Vej 7H, 9220 Aalborg, Denmark; 2 Lantmännen Unibake Denmark, Oensvej 28, Hatting, 8700 Horsens, Denmark

**Keywords:** Rheology, Characterization and analytical techniques, Rheology, Mechanical properties

## Abstract

Alveograph analysis is an established method for flour characterisation, and several alveograph parameters have been introduced over the years. Typically, ten parameters are found for every analysis from the air pressure curve in the modern versions of the alveograph, but the relationships between the parameters and their potential redundancy are not well described in the literature. In this work, an overview of the parameters is provided, including how they are found and what they may represent, and the integral relationship between the parameters was investigated using Pearson correlation analysis of the parameters from 532 pressure curves. The parameters G (swelling index), Dmax (maximum of first derivative), SH (strain hardening index) and K (strength coefficient) exhibited very strong correlations with other alveograph parameters (*r* > 0.97), and these parameters do therefore not provide additional information. The parameters P (maximum overpressure), L (abscissa at rupture), W (deformation energy), P/L (configuration ratio), Ie (elasticity index) and Dmin (minimum of first derivative) on the other hand, represent a relatively basic set of parameters that uniquely characterises various parts of the pressure curves and thus the dough rheology/physics during dough inflation. Nevertheless, even between this basic set of parameters relatively strong correlations were found, signifying that they are interrelated, as they all are affected by changes in the dough constituents.

## Introduction

The alveograph is an empirical tool used to assess the baking quality of wheat flour. The alveograph measures viscoelastic properties of a dough bubble while it is inflated, as the alveograph method attempts to mimic the bubble growth taking place during dough fermentation and in the beginning of baking^1^. The results from the alveograph is widely used for commercial benchmarking of wheat flour and decision making^2^. The alveograph is manufactured by Chopin Technologies. Another device for bubble inflation analysis of dough is the D/R Dough Inflation System (Stable Micro Systems)^3^. It operates by the same principle as the alveograph, however, the analysis conditions are often different, and the results are therefore not directly comparable^4,5^. This work focuses on the alveograph and its related parameters.

In the alveograph standard protocol, a dough is prepared from flour, water and sodium chloride in the integrated mixer using fixed water content (which implies a variable dough consistency), after which five dough pieces are extruded from the mixer, shaped to flat discs of similar size and rested for a fixed period of time. The dough disc is clamped in the sample holder and inflated with air from a central hole in the base, which results in an expanding dough bubble. The dough bubble is inflated until rupture. The inflation pressure is measured as a function of time, and an average curve is made from the five pressure curves. From this curve a range of different parameters are found.

Different versions of the alveograph has been produced over the years, since Marcel Chopin in the 1920s started the development of the apparatus which later was developed into what is known today as the alveograph^1^. Several improvements have been made to the alveograph method, including data collection and parameter calculation. In the older versions of the alveograph, the pressure curve from the bubble inflation was recorded by a manometer using a pen on chart paper, after which the alveograph parameters were calculated manually. Later, the recording manometer was replaced by the alveolink recorder-calculator which automatically collected the data, visualised the pressure curves and calculated the alveograph parameters. In the newer versions, the alveograph is connected to a computer, and dedicated software collects and visualises the data and calculates the alveograph parameters.

**Figure 1:**
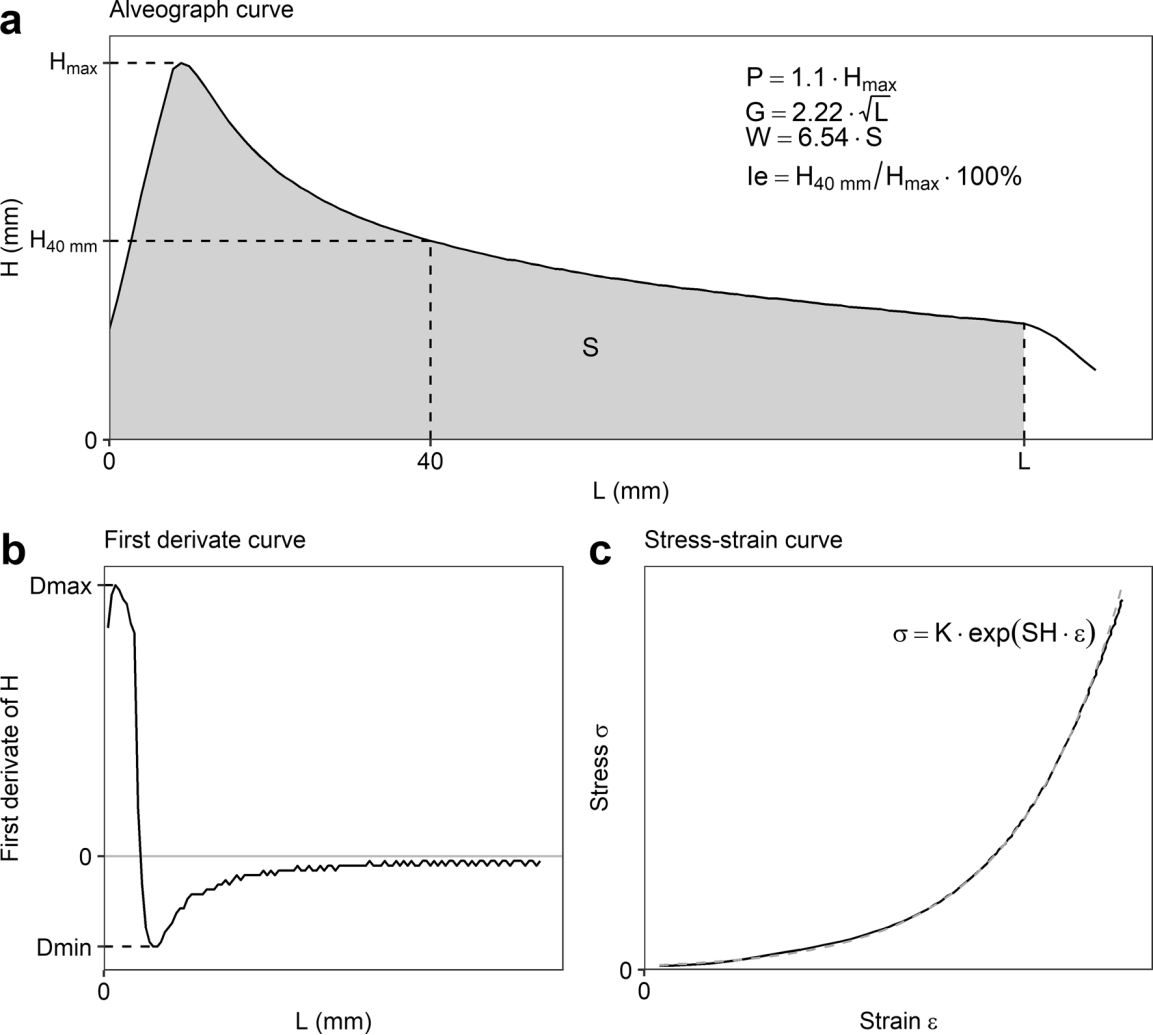
An overview of how the different alveograph parameters are found. **(a)** The alveograph curve from which P, L, G, W and Ie are found. Note, different formulas for calculation of G and W exists. H_max_ is the maximum height of the curve, H_40 mm_ the height of the curve at 40 mm on the L axis and S the area under the curve. **(b)** The first derivate curve from which Dmin and Dmax are found. **(c)** The stress–strain curve calculated as described by Bloksma^15^ from which SH and K are found by fitting of an exponential equation.

Over the years, the number of alveograph parameters extracted from the pressure curve, see Fig. 1, has increased. Traditionally, the parameters from analog output on chart paper have been based on the height and length of the curve and the area under the curve, yielding a set of parameters including maximum overpressure (P), average abscissa to rupture (L), swelling index (G), configuration ratio (P/L) and deformation energy (W)^1^. These have traditionally been and are still the most used parameters^1^. With the introduction of computers and modern data treatment, new parameters based on the shape of the curve, such as the elasticity index (Ie), and the first derivative curve, providing minimum and maximum of first derivative (Dmin and Dmax), were established^6,7^. The pressure curve has also been recalculated into a stress–strain curve, from which other parameters, such as the strain hardening index (SH) and the strength coefficient (K), were estimated^4^. In total, ten parameters can be provided for each analysis today (Alveolab user manual, Chopin Technologies, 12/2016). Some of the parameters extracted from the alveograph pressure curves are widely used, while other parameters have only been infrequently reported and remain less described. A critical review of the parameters is missing in the literature, and it is less obvious how the various parameters relate to each other.

The aim of this work is to investigate whether and eventually which of the alveograph parameters that are closely related and thereby represent the same information. For that purpose, Pearson correlation analysis is used. An overview of the alveograph parameters is also provided, including how they are determined, what they represent, and which material properties they are known to be related to.

## Theory

### Maximum overpressure, P

The parameter P is the maximum overpressure needed to inflate the dough bubble measured in mm H_2_O. The parameter is also called the dough tenacity. P is found from the maximum height of the average curve measured in mm multiplied by a factor of 1.1 (due to the geometry of the water manometer initially used)^1^. P is one of the most used alveograph parameters, although the interpretation of it is also one of the most debated. It is considered to be the indicator of dough resistance to deformation^1,8^, but it has also been claimed that it is an indicator of dough tensile strength in the initial stage of deformation^9^, related to the stiffness, shortness and tightness of the dough^10,11^, an indicator of dough stability^12^, and related to the viscosity of the dough and water-absorbing capacity of the flour^13^. On the other hand, when the alveograph curve is recalculated into a stress–strain curve, it is observed that P has no significance in this curve, as there is no inflection coinciding with the maximum overpressure^14,15^. This has been explained by Bloksma^15^, who describes that the maximum overpressure during bubble inflation originates from the crossover point between the increasing stress and the increasing surface area, and the maximum overpressure is therefore based on the geometry of the experiment. Despite P has no significance in terms of fundamental rheology, the parameter is widely used.

P has been found to be related to the water absorption of the flour, as increasing levels of damaged starch and amount of middlings and bran, which are all known to increase the water absorption, have been shown to increase P^16–19^. On the other hand, the water content in the dough is negatively related with P^18,19^. Furthermore, it is generally accepted that P is to some degree dependent on the gluten network in the dough^20–22^. However, P does not seem to be strongly correlated with the flour protein content, as most studies find no or a weak (positive or negative) correlation between P and protein content^7,23–26^.

### Average abscissa to rupture, L

The parameter L is the length of the average curve from start of inflation until rupture of the dough bubble (which is observed as a sudden drop in pressure) measured in mm, as seen in Fig. 1a. L corresponds to time using a conversion factor of 5.5 mm/s (originating from the speed of the recording drum used in the early versions of the alveograph)^1^. L is also called the dough biaxial extensibility or just extensibility, as it is a measure of how much the dough can be extended before it breaks.

Biaxial extensibility is an important parameter in relation to the baking quality, as a sufficiently large extensibility is required to prevent premature rupture of dough membranes between gas cells to obtain large bread volume and fine crumb structure^27^. The extensibility of a dough is dependent on both the extension rate due to viscous flow, as well as the elastic properties of the dough influence the amount of stress required to stretch the dough^28,29^. The extensibility of a dough is also related to the strain hardening properties, as the strain hardening stabilises the dough membrane during extension^14,30^. Extensibility of wheat flour doughs is among other things associated with the structure of the gluten network and the molecular size distribution of the gluten polymers, as polymer entanglement network theory has been suggested to explain extensibility on a molecular level^29^. Extensibility has also been shown to be affected by multiple variables besides the gluten network quality, including the water absorption capacity of the flour and the water content in the dough^17–19^. Multiple studies have shown that L has strong, positive correlations with several bread properties, including bread volume^7,23,31,32^.

### Swelling index, G

The swelling index G is defined as the square root of the average volume of air in ml used for inflating the dough until rupture^1^. G is another measure of the extensibility, but it has also been stated that G is related to the spring and the shortness of the dough^13^.

G is calculated from L, as it is assumed that the volume of the dough bubble increases uniformly with time^1^. The swelling index can be calculated using the formula $G=2.226\cdot \sqrt{L}$ ^33^. However, according to the AlveoLab user manual (Chopin Technologies, 12/2016) the swelling index is calculated by $G=2.22\cdot \sqrt{L}$.

As G is calculated from L, it is dependent on and affected by the same variables as L. However, some minor differences in correlation coefficients and *p*-values in significance tests might be found between L and G (see e.g. Cappelli et al.^19^, Huen et al.^34^ and Rasper et al.^26^), as G is not linear dependent on L, but found from the square root of L.

### Deformation energy, W

The parameter W is called the deformation energy, as it represents the energy required to inflate the dough bubble until rupture^1^. It is sometimes referred to as flour strength, dough strength, baking strength or flour protein strength. W is one of the industrially most applied alveograph parameters, as it is used for prediction of processing behaviour of flour cultivars^1,33^. W can be used to divide different cultivars into groups with different strength^35^. In flour specifications for different types of bakery products, W can be applied (possibly together with P/L), as e.g. bread flours are characterized by larger W values compared to biscuit flours^36^. W is derived from the area under the curve, and it is normally provided in the unit 10^–4^ J.

For the standard alveograph analysis, the deformation energy in 10^–4^ J can be calculated using the formula $W=6.54\cdot S$, in which S is the area under the curve measured in cm^2^ ^1,26^, while the AlveoLab user manual (Chopin Technologies, 12/2016) states it is calculated by $W=1.32\cdot \frac{V}{L}\cdot S$, in which V is the volume of air measured in ml. As W is calculated from the area under the curve, it is influenced by P and L, and changes in W will therefore also be a reflection of changes in P and L^23,24,37^. Preston et al.^18^ notice that W is normally more influenced by changes in P than in L.

W is generally accepted to be an indicator of gluten strength, as it has been shown to be dependent on the amount and quality of the gluten in the dough^20,24,38^. However, W is also dependent on other variables, as the alveograph analysis is made on dough and not on gluten. W is positively related to the water absorption of the flour^17,18^, while higher hydration decreases W^18,19^. Positive, significant correlations (*p* < 0.05) have previously been found between W and bread volume^7,23,31,32^.

### Configuration ratio, P/L

The configuration ratio P/L is the ratio between the maximum overpressure and the length of the curve. High P/L indicates a resistant and inextensible dough, while low P/L indicates a weak and extensible dough. The value is dimensionless. P/L is often used industrially together with W to assess flour quality, as P/L indicates the shape of the alveogram and thereby the balance between tenacity and extensibility. However, while the magnitude of W is used to assess which types of bakery products a flour is appropriate for, the requirements to P/L are often constant and independent of the area of application^33^. Abuhammad et al.^35^ found that P/L could not be used to discern between flour qualities unless the flours had high W values, which resulted in high P/L. Although P/L is simple to calculate, it is not as frequently used in the literature as the above mentioned alveograph parameters.

### Elasticity index, Ie

The parameter Ie is called the elasticity index, and it was defined by Kitissou^6^. Ie is calculated as stated in Eq. (1), as it is the ratio between the pressure 40 mm from the start of the curve on the L-axis and the maximum overpressure (see Fig. 1a), and it is measured in percentage. The elasticity index can therefore be calculated from the height of the pressure curve at 40 mm divided by the maximum height of the pressure curve. The volume of air injected at a curve length of 40 mm is approximately 200 ml, and the pressure at this point is therefore sometimes referred to as P_200_. The use of Ie is limited when working with very inextensible doughs, as it is not possible to calculate Ie, if the curve has a length below 40 mm.

1 $$Ie = \frac{{P_{200} }}{P} \cdot 100 \% = \frac{{H_{40 mm} }}{{H_{max} }} \cdot 100 \%$$

The pressure needed to inflate the dough bubble is dependent on both the dough properties and the dough thickness, which decreases during the analysis. However, it is assumed when 200 ml of air is injected that the resistance to deformation and thereby the pressure is mainly linked to the strength of the internal bonding forces and not so much the thickness of the dough^6^. The ratio between the pressure at this point and the maximum pressure can therefore be used as an indication of the dough elasticity according to Kitissou^6^.

Ie has been found to be affected by different flour constituents as well as addition of different ingredients^6^. The elasticity index of a flour needs to be within a certain range, dependent on the type of product. According to Kitissou^6^, too high and low values of Ie are not desirable, as e.g. doughs with high Ie tend to be hard to elongate and shrinks. Comparisons of Ie and bread properties are limited in the literature, but Duyvejonck et al.^39^ found a significant, positive correlation (*p* < 0.05) between Ie and bread volume. Huen et al.^34^ found that Ie was positively correlated with the height/width ratio for rolls.

### Minimum of first derivative, Dmin

The first derivative curve can be found from the alveograph curve, and the minimum value on this curve is the parameter Dmin as seen in Fig. 1b. Dmin corresponds to the steepest slope of the curve, when the pressure decreases (after the maximum overpressure). The parameter was first defined by Addo et al.^7^. However, Dmin is reported as a negative value in the alveograph software, while Addo et al.^7^ defined Dmin (or actually DM) as the negative of the minimum value in order to obtain a positive value.

Addo et al.^7,40^ found that Dmin had a significant correlation (*p* < 0.05) with the bread volume, as the closer Dmin was to zero, the higher the bread volume in general. Furthermore, the effect of defatting of the flour^37^ and addition of shortening and sucrose esters^41^ on Dmin have been studied. These are some of the only uses of the Dmin in the literature.

### Maximum of first derivative, Dmax

The parameter Dmax is the maximum value of the first derivative of the alveograph curve. It corresponds to the steepest slope of the curve, when the pressure increases (before the maximum overpressure), and Dmax is therefore a positive value.

There is no description of Dmax in the literature to the best of the authors’ knowledge. In the article from which Dmin was found, they did not observe this maximum on the first derivative curve^7^.

### Strain hardening index, SH

The parameter SH is the strain hardening index. Strain hardening is defined as the phenomenon that the stress required to deform a material increases more than proportionally to the strain (at increasing strain and constant strain rate)^30^.

The strain hardening index is found by fitting the empirical exponential equation in Eq. (2) ^4^, in which σ is the stress, ε the Hencky strain, SH the strain hardening index and K the strength coefficient (see next section), to the stress–strain curve (Fig. 1c).

2 $$\sigma = K \cdot \exp \left( {SH \cdot \varepsilon } \right)$$

The stress–strain curve is calculated from the alveograph curve by the use of equations proposed by Bloksma^15^. The stress and the strain are calculated for the pole of the dough bubble based on different assumptions^15^. These assumptions have however been shown not to be valid at the conditions stated in the standard alveograph protocol, which results in that the stress and to a lesser extent the strain are overestimated^42–44^ and thereby resulting in a too high SH. Furthermore, the strain rate is not constant, as the air flow rate is constant, which result in decreasing strain rate at the pole of the bubble during inflation^43^. It has been shown that dough exhibits both strain hardening and strain rate hardening, which is stiffening of the dough at higher deformation rates, and the strain hardening index is therefore also dependent on the strain rate^14,45^. SH is therefore only the apparent strain hardening index.

Strain hardening index can be found both for uniaxial and biaxial extension, but the obtained index values are different^46^. Different methods for determination of strain hardening index in biaxial extension may also lead to different results, as the prerequisites are not always fulfilled, including difficulties in accurate determination of strain and stress, variable strain rates, as well as the use of different equations for estimation of the strain hardening index^47^. As the strain hardening index is dependent on the method, we have decided to focus only on results acquired using bubble inflation method in the following.

Only few studies determine SH for conditions similar to alveograph standard analysis. Several studies use a D/R Dough Inflation System, which is often operated at an approximate constant strain rate of 0.1 s^−1^ ^4^, while the alveograph analysis with a constant air flow rate entail a higher, variable strain rate. The type of deformation rate (constant strain rate or constant flow rate) influences SH, as demonstrated by Chin et al.^5^, and the results are therefore not directly comparable.

It is generally accepted that strain hardening is related to the properties of the gluten network, as strain hardening has been explained on a molecular level by entanglement coupling of large glutenin molecules^29,48^. It is therefore assumed that strain hardening is dependent on the number of branches and the entanglement of the gluten polymers, which again is primarily dependent on molecular size distribution of the proteins^49^. SH has been shown to be affected by the strength and development of the gluten network^5,50,51^.

A high strain hardening index was first proposed to be important for the performance of a bread dough by van Vliet et al.^30^, as strain hardening facilitates inflation of the dough bubbles to larger volumes and with thinner cell walls before failure by locally increasing the resistance to deformation^14^. Multiple studies have found a correlation between bread volume and SH^4,49,52^.

### Strength coefficient, K

The parameter K is denoted the strength coefficient. It is found together with SH, as it is the coefficient in the exponential equation in Eq. (2), and K is therefore also known as the strain hardening coefficient. The exponential equation fitted is an empirical equation, as already noted in the previous section, and K is therefore a fitting constant approximating the stress when the strain equals 0. An increase in K indicates a more viscous or stiffer dough^5^. Compared to SH, K is rarely applied. This is possibly caused by the less clear interpretation of K, since it is assumed to be related to both the viscous and elastic properties of the dough^5^.

Different challenges are linked to the determination of the stress–strain curve and the strain hardening index (see above section), and these also apply to the strength coefficient, which might result in different values of K. Furthermore, many of the results for K obtained at bubble inflation are found at conditions different from the standard alveograph analysis affecting the value of K^5^.

## Results and discussion

**Figure 2:**
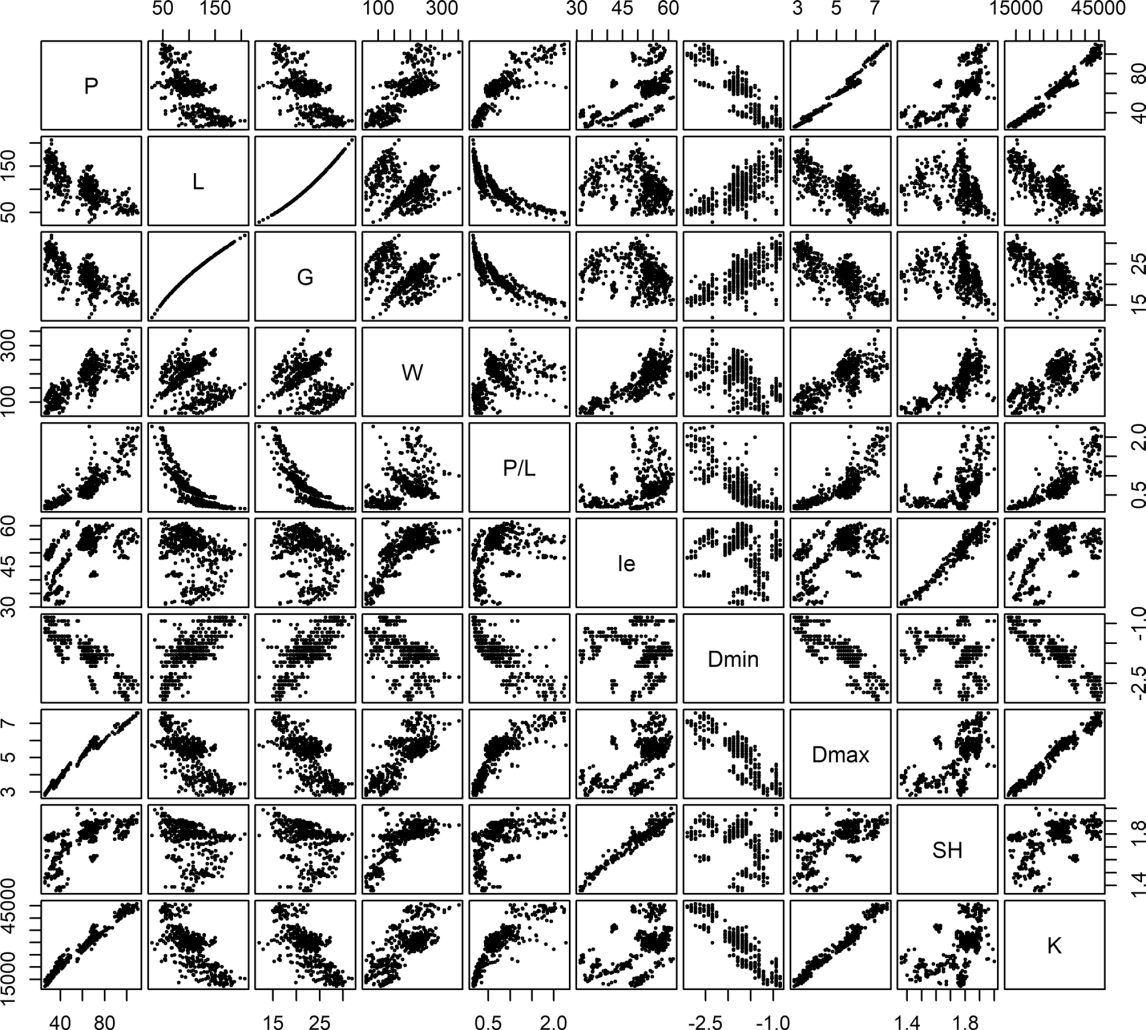
Scatter plot matrix between the different alveograph parameters.

The results for different test series analysed with alveography were used to compare the ten alveograph parameters. The mean, range and the coefficient of variation (CV) of the alveograph parameter values are given in Table 1. Each of the alveograph parameters covers a wide range of values and is thus considered to be representative of the diversity of experimental results obtained from the alveograph pressure curves. The dataset is therefore judged to be suitable for uncovering the integrated associations of the alveograph parameters. The alveograph parameters for the alveograph curves were compared, and the pairwise scatter plots can be seen in Fig. 2, while Table 2 shows the Pearson correlation coefficients between the alveograph parameters. In the scatter plots in Fig. 2, clusters of data points can be observed between some of the parameters, e.g. for P and W, L and W, P and Ie as well as SH and K. Further investigation of these reveal that these clusters do partly originate from different dough compositions (from the ingredients added to the dough) and to a lesser extent the flour qualities (see Supplementary Fig. S1 and S2). All the correlations between the parameters in Table 2 were significant with a *p*-value < 0.01. Some of the correlations between the alveograph parameters are discussed below.

**Table 1:** Mean, range and coefficient of variation (CV) for the ten alveograph parameters.

	Mean	Range	CV (%)
P (mm H_2_O)	60.5	26–110	33.8
L (mm)	105.1	29–207	31.7
G	22.5	12.0–31.9	16.2
W (10^–4^ J)	182.4	62–352	31.4
P/L	0.70	0.15–2.28	65.4
Ie (%)	50.9	31.1–61.5	14.4
Dmin	−1.72	− 2.91 to − 0.85	− 26.7
Dmax	5.11	2.82–7.61	23.2
SH	1.76	1.36–2.00	7.5
K	28,206.3	12,270–45,780	29.0

**Table 2:** Pearson correlation coefficients between the different alveograph parameters. All correlations were significant with a *p*-value < 0.01.

	P	L	G	W	P/L	Ie	Dmin	Dmax	SH
L	− 0.74								
G	− 0.74	0.99							
W	0.76	− 0.25	− 0.22						
P/L	0.87	− 0.85	− 0.88	0.39					
Ie	0.57	− 0.24	− 0.23	0.81	0.36				
Dmin	− 0.85	0.71	0.70	− 0.49	− 0.79	− 0.11			
Dmax	0.98	− 0.74	− 0.73	0.78	0.83	0.59	− 0.85		
SH	0.62	− 0.28	− 0.28	0.76	0.46	0.97	− 0.17	0.61	
K	0.98	− 0.76	− 0.75	0.72	0.85	0.46	− 0.91	0.98	0.49

The maximum overpressure P and the length of the curve L were significantly, negatively correlated, which was also observed by Bettge et al.^23^ and Zanetti et al.^53^, while Addo et al.^7^, García-Álvarez et al.^36^ and Edwards et al.^24^ did not find any significant correlation between these two parameters. The negative correlation between P and L might be explained by the choice of test series, as for specific test series (e.g. fixed flour quality and changing one variable) it was observed that these parameters were affected in opposite directions. When comparing the different dough compositions and flour qualities, a higher independency between P and L was observed in some cases, while similar correlation coefficients was found in other cases.

The swelling index G was almost perfectly correlated with L as expected, as G is calculated from L. The correlation coefficients for L and G versus the other parameters were therefore almost identical. The correlation between G and L was not linear as observed in Fig. 2, as G is found from the square root of L.

The deformation energy W was significantly, positively correlated with P, which was also observed by multiple other studies^7,23,24,36,53^. As clusters were observed in the scatter plot with P and W in Fig. 2, the correlation coefficients for P and W for the doughs with different ingredients were compared. These correlation coefficients were in general lower, but positive (see Supplementary Table S4–S7). W is calculated from the area under the curve, and it is therefore influenced by the height of the curve, which P is obtained from. A weak, negative correlation was observed between W and L. In the literature, the correlation coefficients were in general lower for L and W compared to P and W, but a positive correlation was generally found^7,23,24,36,53^. However, on Fig. 2 it appeared that several clusters exhibit a positive correlation between L and W, which intuitively may be expected to originate from different dough compositions or flour qualities. This was confirmed when the doughs with different ingredients were compared, as positive moderate or strong correlations for L and W were found for the individual dough compositions, while negative correlations between L and W were found for the different flour qualities (see Supplementary Table S1–S7).

The configuration ratio P/L was positively correlated with P and negatively correlated with L as expected, as larger P values will result in a higher ratio, while higher L values will decrease the ratio. Similar correlations were observed by García-Álvarez et al.^36^ and Zanetti et al.^53^. These correlations were not linear as seen in Fig. 2, as the P/L ratio is less affected by changes at high L values and low P values.

The elasticity index Ie had a strong positive correlation with W, which was also observed by Zanetti et al.^53^. In principal component analysis variable projection plots, Ie was often located in the proximity of W compared to the other alveograph parameters^33,39,54^, indicating that they were positively correlated. However, Kitissou^6^ stated that Ie appeared to be independent of W and suggested that Ie represents substantial additional information.

The minimum of first derivative Dmin was negatively correlated with P, positively correlated with L and negatively correlated with W. In comparison, Addo et al.^7^ found no significant correlation (*p* < 0.05) between Dmin and P and a positive correlation with L as well as with W, when comparing different qualities of flour. The negative correlation between Dmin and P might be partly explained by the shape of the curve where a higher maximum height will imply a steeper decreasing slope. The discrepancy between our results and Addo et al.^7^ may be explained by the choice of test series, as Addo et al.^7^ compared different flour qualities, while we have in this work only analysed few flour qualities and to a higher extent varied analysis conditions and added different ingredients. This may also explain the reverse correlation between Dmin and W, as it has previously been shown that Dmin and W were affected in opposite directions for some different treatments of one type of flour^37^.

The maximum of first derivative Dmax was almost perfectly positively correlated with P. Dmax is the steepest slope at the increasing pressure in the beginning of the analysis, and it is therefore probable that the higher the P value, the higher the slope. Other strong correlations between Dmax and other alveograph parameters can also be found, but these can be explained by their correlation with P.

The strain hardening index SH was close to perfectly positively correlated with Ie. SH indicates the rate of increase of the exponential equation, which is fitted to the stress–strain curve calculated from the alveogram. The calculation of the stress–strain curve is only dependent on the inflation time and the pressure (assuming constant air flow and initial sample geometry)^15,45^, and the stress–strain curve and the alveogram are therefore dependent on the same variables despite the different shapes of the curves. SH indicates the shape of the stress–strain curve, while Ie indicates the shape of the alveogram based on the maximum height of the curve and the height of the curve at 40 mm on the L axis, and this might explain the very strong correlation between them. No comparisons of Ie and SH could be found in the literature to the best of the authors’ knowledge. SH was strongly, positively correlated with W and P, as seen in Table 2. Likewise, Tronsmo et al.^55^ found SH located close to P and W in principal component analysis variable projection plot, indicating a positive correlation, while Bollaín & Collar^56^ found a positive correlation between SH and W, but no significant correlation (*p* < 0.05) between SH and P. A weak, negative correlation between SH and L was observed in Table 2. However, multiple studies have found positive correlations between strain hardening and failure strain (the strain at which the bubble ruptures)^4,5,14,49,51,52^, and it is clear that strain hardening is important for the extensibility of the dough. As the calculation of strain is only dependent on inflation time, failure strain and L is closely correlated^56^. The weak negative correlation between SH and L was therefore not expected. However, when the correlation coefficients between SH and L were calculated for the different dough compositions, they were either weakly positive or not related, but no negative correlation was found (see Supplementary Table S4–S7). This might also be influenced by differences in the experimental protocol, as the studies mentioned above used other flow rates than the alveograph standard method.

The strength coefficient K was nearly perfectly positively correlated with P. K designates the intercept with the stress axis in the exponential equation fitted to the stress–strain curve, from which SH also is found. Like K indicates the stress at low strain, P indicates the pressure in the beginning of the curve, which might explain the high correlation between them. K and SH appeared to be positively correlated. However, multiple clusters can be observed in the scatter plot with K and SH in Fig. 2, which is at least partly caused by different dough compositions and flour qualities. When the correlation coefficients were calculated for the individual dough compositions, no correlation was found between K and SH, while positive correlation coefficients are found for the individual flour qualities (see Supplementary Table S1–S7). In the literature, it was also observed that the SH and K were correlated, however, a positive or negative correlation was found dependent whether different flour qualities or dough formulations were compared^5,51,55^. K and SH are found from the same equation, which might explain why are correlated, at least in some cases.

The parameters P, Dmax and K all describe the behaviour of the dough at the beginning of the inflation, a probable explanation to why they are closely correlated. Other parameters describe the shape of the curve (after the maximum overpressure). This includes Ie and SH, which are also almost perfectly correlated, as well as Dmin, which are only weakly correlated with Ie and SH, but strongly correlated with others of the alveograph parameters. These three parameters are found from different parts of the pressure curve, as Dmin are found close to the maximum overpressure, while Ie and SH are also based on data further away from the maximum overpressure. W and P/L are both to some degree dependent on P and L. However, as W is partly related to the product of P and L, and P/L is defined as the ratio between the two factors, the correlation between them is therefore weak.

## Conclusion

In summary, the parameters G, Dmax, SH and K had correlation coefficients of 0.97 or higher with L, P, Ie and P, respectively. These parameters do therefore not contain additional information which is not covered in the other parameters. P, L, W, P/L, Ie and Dmin represent a basic set of parameters for the characterisation of the pressure curves, although relatively strong correlations can be found between some of them. This is not surprising, as they are obtained from the same curve and therefore may not be considered as truly independent parameters. A given pressure curve is affected by changes in dough constituents, e.g. the quality of the gluten network or the water content, and specific changes in one factor of the dough will therefore cause changes of in several of the alveograph parameters to various degrees. This means that the six basic alveograph parameters are to a greater or lesser extent interlinked, although the details of the links between them are currently unknown or diffuse at best. Further research on the links between the alveograph parameters and what they signify with regard to the physics of the inflating dough bubble and its intrinsic properties is therefore needed.

## Methods

For the comparison of the alveograph parameters, alveograph analysis results from a range of different test series were used. In the standard alveograph method, a dough was made from wheat flour and sodium chloride solution. The dough was mixed for 8 min, after which five dough pieces were extruded. The dough pieces were rolled and cut out to similar sized dough discs and rested. 20 min after the mixing stopped, the dough discs were analysed one at a time by inserting the dough disc into the alveograph. A bubble was inflated until rupture, and the pressure in the bubble was measured as a function of time. From this curve, multiple parameters were found. Some of the test series were performed under condition as described by the standard AACC method 54-30.02^8^, while other were performed at lower analysis temperature, different mixing time, variable water addition and/or with the addition of different ingredients. Three different qualities of wheat flours (intended for Danish pastry, bread and cake) and different batches of one of them were analysed. A total of 41 different experiments were analysed in triplicate. An overview of the different test series, including type of flour, eventually added ingredients and deviations from the standard protocol, can be seen in Supplementary Table S8.

The analyses were performed on an AlveoLab (Chopin Technologies, Villeneuve-la-Garenne, France) with the AlveoLab software version 1.1.1.14. The results for parameters P (mm H_2_O), L (mm), G, W (10^–4^ J), P/L, Ie (%), Dmin, Dmax, SH and K were exported from the software. As large variation was observed between the five alveograph curves for each dough for some of the test series (coefficients of variation up to 27% for P, 51% for L and 33% for W), the alveograph parameters for the individual curves and not the average curve were applied in this work. For some of the curves, it was observed that the bubble inflation was terminated before the bubble ruptured. This occurred sometimes due to an error in the software if the pressure was very low. These curves were omitted from the dataset. Ie values of 0, which was obtained for curves with L below 40 mm, was also removed before data analysis. This resulted in 532 individual curves (see Supplementary Table S8), and the parameters from those were applied for the statistical analysis. Scatter plots and Pearson correlation coefficients were obtained using R version 3.6.1.

## Supplementary Information

Supplementary Information.

## Data Availability

Requests for materials should be addressed to K.L.L.
