# Supplementary material for: Investigation of the relationships between the alveograph parameters
Source: Sci Rep. 2021 Mar 5;11:5349. doi: 10.1038/s41598-021-84959-3 (PMC7935897; doi:10.1038/s41598-021-84959-3)
Supplement: Supplementary file 1 — Supplementary Information. [file 41598_2021_84959_MOESM1_ESM.docx]

**Supplementary information**

Investigation of the relationships between the alveograph parameters

Anne-Sophie Schou Jødal^1,2^ and Kim Lambertsen Larsen^1^*

^1^Department of Chemistry and Bioscience, Section of Chemistry, Aalborg University, Fredrik Bajers Vej 7H, DK-9220 Aalborg, Denmark

^2^Lantmännen Unibake Denmark, Oensvej 28, Hatting, DK-8700 Horsens, Denmark

* Corresponding author, kll@bio.aau.dk

Scientific Reports

**Supplementary Figure S1.** Scatter plot matrix between the different alveograph parameters coloured according to flour quality.

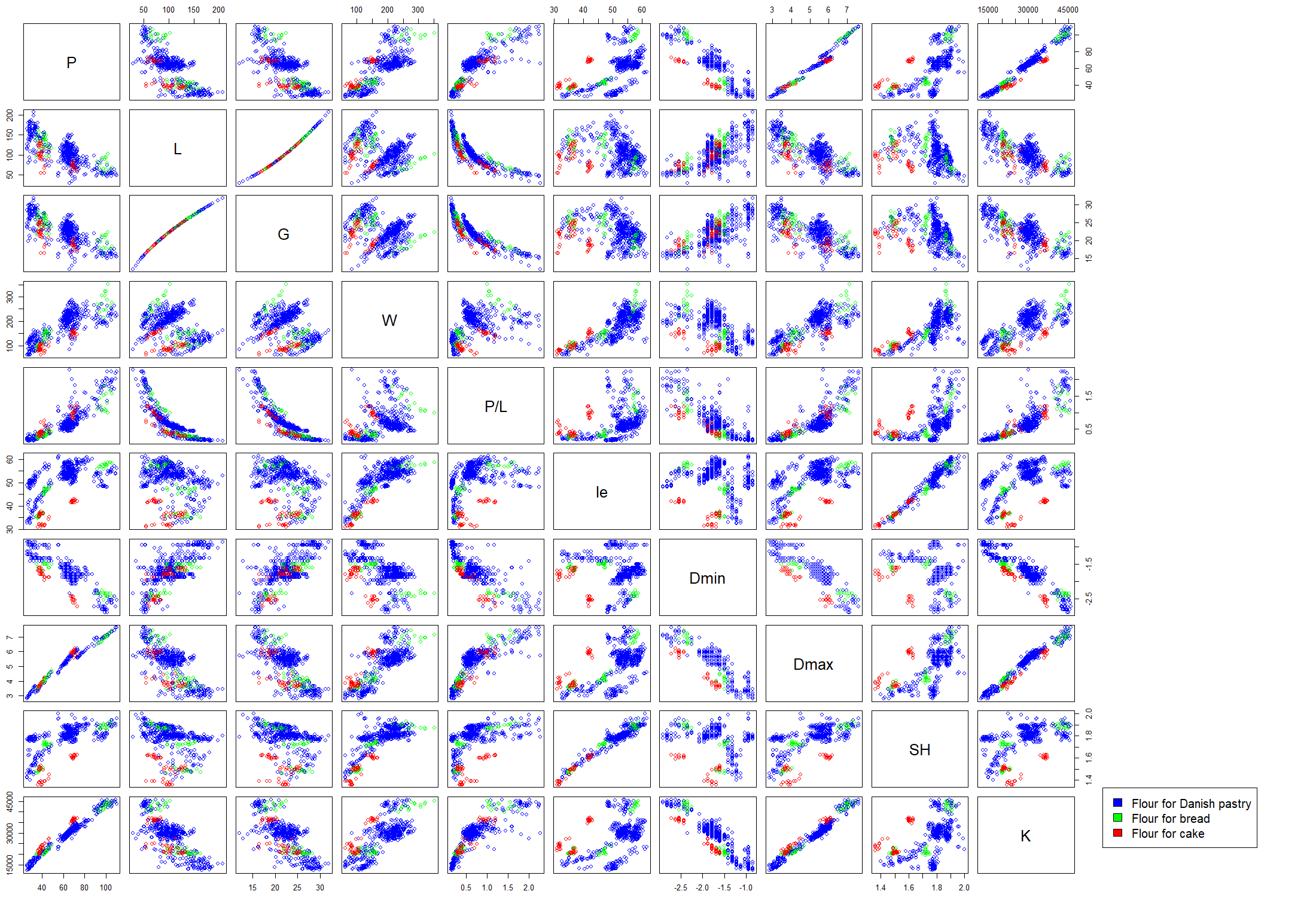


**Supplementary Figure S2.** Scatter plot matrix between the different alveograph parameters coloured according to dough composition (dependent on the ingredients added).

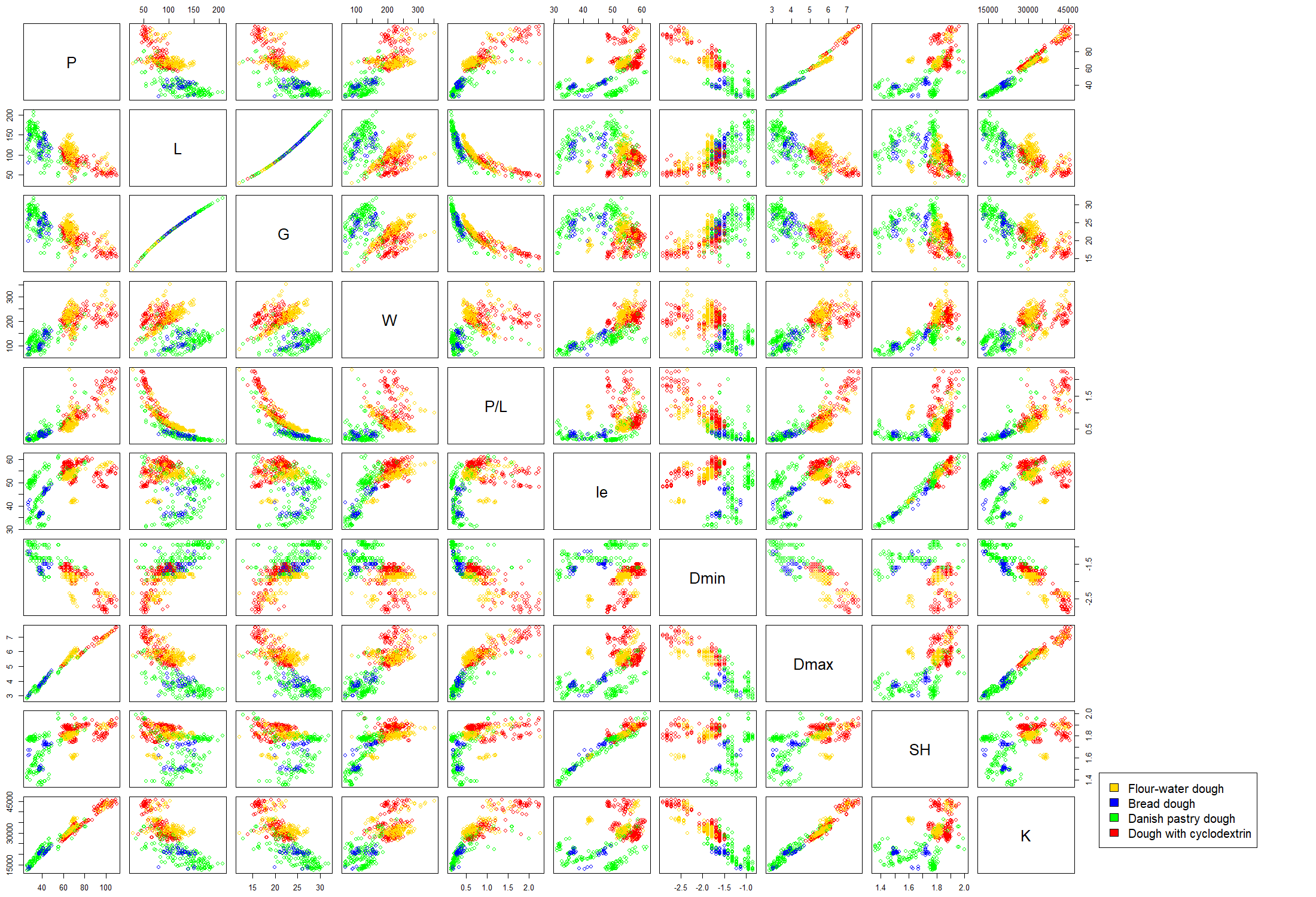


**Supplementary Table S1.** Correlation coefficients between the different alveograph parameters for wheat flour intended for Danish pastry dough.

|  | P | L | G | W | P/L | Ie | Dmin | Dmax | SH |
| --- | --- | --- | --- | --- | --- | --- | --- | --- | --- |
| L | -0,78 |  |  |  |  |  |  |  |  |
| G | -0,77 | 0,99 |  |  |  |  |  |  |  |
| W | 0,73 | -0,27 | -0,23 |  |  |  |  |  |  |
| P/L | 0,87 | -0,86 | -0,89 | 0,32 |  |  |  |  |  |
| Ie | 0,54 | -0,35 | -0,33 | 0,75 | 0,32 |  |  |  |  |
| Dmin | -0,91 | 0,70 | 0,70 | -0,59 | -0,81 | -0,22 |  |  |  |
| Dmax | 0,98 | -0,77 | -0,75 | 0,77 | 0,82 | 0,57 | -0,90 |  |  |
| SH | 0,63 | -0,42 | -0,42 | 0,68 | 0,49 | 0,95 | -0,34 | 0,63 |  |
| K | 0,99 | -0,77 | -0,76 | 0,73 | 0,83 | 0,49 | -0,94 | 0,98 | 0,55 |

**Supplementary Table S2.** Correlation coefficients between the different alveograph parameters for wheat flour intended for bread.

|  | P | L | G | W | P/L | Ie | Dmin | Dmax | SH |
| --- | --- | --- | --- | --- | --- | --- | --- | --- | --- |
| L | -0,85 |  |  |  |  |  |  |  |  |
| G | -0,85 | 1,00 |  |  |  |  |  |  |  |
| W | 0,95 | -0,68 | -0,68 |  |  |  |  |  |  |
| P/L | 0,96 | -0,91 | -0,93 | 0,82 |  |  |  |  |  |
| Ie | 0,90 | -0,73 | -0,73 | 0,93 | 0,83 |  |  |  |  |
| Dmin | -0,97 | 0,85 | 0,86 | -0,87 | -0,95 | -0,78 |  |  |  |
| Dmax | 1,00 | -0,85 | -0,85 | 0,95 | 0,95 | 0,92 | -0,96 |  |  |
| SH | 0,85 | -0,69 | -0,70 | 0,88 | 0,80 | 0,99 | -0,72 | 0,87 |  |
| K | 0,99 | -0,84 | -0,84 | 0,94 | 0,94 | 0,86 | -0,98 | 0,99 | 0,79 |

**Supplementary Table S3.** Correlation coefficients between the different alveograph parameters for wheat flour intended for cake.

|  | P | L | G | W | P/L | Ie | Dmin | Dmax | SH |
| --- | --- | --- | --- | --- | --- | --- | --- | --- | --- |
| L | -0,67 |  |  |  |  |  |  |  |  |
| G | -0,67 | 1,00 |  |  |  |  |  |  |  |
| W | 0,93 | -0,36 | -0,36 |  |  |  |  |  |  |
| P/L | 0,93 | -0,86 | -0,87 | 0,74 |  |  |  |  |  |
| Ie | 0,87 | -0,34 | -0,34 | 0,95 | 0,72 |  |  |  |  |
| Dmin | -0,99 | 0,74 | 0,74 | -0,87 | -0,95 | -0,80 |  |  |  |
| Dmax | 0,99 | -0,69 | -0,69 | 0,91 | 0,93 | 0,85 | -0,98 |  |  |
| SH | 0,81 | -0,30 | -0,31 | 0,89 | 0,68 | 0,98 | -0,73 | 0,78 |  |
| K | 0,99 | -0,71 | -0,70 | 0,89 | 0,93 | 0,80 | -0,99 | 0,99 | 0,72 |

**Supplementary Table S4.** Correlation coefficients between the different alveograph parameters for doughs with flour, water and sodium chloride.

|  | P | L | G | W | P/L | Ie | Dmin | Dmax | SH |
| --- | --- | --- | --- | --- | --- | --- | --- | --- | --- |
| L | -0,43 |  |  |  |  |  |  |  |  |
| G | -0,43 | 1,00 |  |  |  |  |  |  |  |
| W | 0,41 | 0,61 | 0,62 |  |  |  |  |  |  |
| P/L | 0,70 | -0,87 | -0,90 | -0,30 |  |  |  |  |  |
| Ie | 0,30 | 0,30 | 0,31 | 0,73 | -0,09 |  |  |  |  |
| Dmin | -0,59 | 0,64 | 0,65 | 0,27 | -0,71 | 0,57 |  |  |  |
| Dmax | 0,95 | -0,48 | -0,47 | 0,32 | 0,70 | 0,16 | -0,66 |  |  |
| SH | 0,33 | 0,21 | 0,21 | 0,64 | 0,01 | 0,98 | 0,51 | 0,18 |  |
| K | 0,94 | -0,49 | -0,49 | 0,25 | 0,69 | -0,02 | -0,79 | 0,94 | -0,02 |

**Supplementary Table S5.** Correlation coefficients between the different alveograph parameters for doughs with addition of either α-, β- or γ-cyclodextrin.

|  | P | L | G | W | P/L | Ie | Dmin | Dmax | SH |
| --- | --- | --- | --- | --- | --- | --- | --- | --- | --- |
| L | -0,77 |  |  |  |  |  |  |  |  |
| G | -0,78 | 1,00 |  |  |  |  |  |  |  |
| W | 0,25 | 0,37 | 0,37 |  |  |  |  |  |  |
| P/L | 0,92 | -0,92 | -0,93 | -0,11 |  |  |  |  |  |
| Ie | -0,37 | 0,51 | 0,52 | 0,51 | -0,50 |  |  |  |  |
| Dmin | -0,91 | 0,76 | 0,77 | -0,02 | -0,88 | 0,70 |  |  |  |
| Dmax | 0,98 | -0,79 | -0,80 | 0,19 | 0,92 | -0,40 | -0,90 |  |  |
| SH | 0,15 | -0,01 | -0,02 | 0,42 | 0,11 | 0,78 | 0,22 | 0,10 |  |
| K | 0,98 | -0,76 | -0,76 | 0,21 | 0,89 | -0,50 | -0,95 | 0,97 | -0,04 |

**Supplementary Table S6.** Correlation coefficients between the different alveograph parameters for doughs with ingredients for Danish pastry.

|  | P | L | G | W | P/L | Ie | Dmin | Dmax | SH |
| --- | --- | --- | --- | --- | --- | --- | --- | --- | --- |
| L | -0,70 |  |  |  |  |  |  |  |  |
| G | -0,72 | 0,99 |  |  |  |  |  |  |  |
| W | 0,53 | 0,12 | 0,10 |  |  |  |  |  |  |
| P/L | 0,88 | -0,80 | -0,85 | 0,22 |  |  |  |  |  |
| Ie | 0,36 | 0,09 | 0,05 | 0,84 | 0,26 |  |  |  |  |
| Dmin | -0,43 | 0,57 | 0,54 | 0,31 | -0,38 | 0,65 |  |  |  |
| Dmax | 0,98 | -0,70 | -0,72 | 0,50 | 0,86 | 0,31 | -0,48 |  |  |
| SH | 0,30 | 0,13 | 0,08 | 0,75 | 0,28 | 0,98 | 0,69 | 0,24 |  |
| K | 0,93 | -0,76 | -0,76 | 0,34 | 0,77 | 0,06 | -0,69 | 0,94 | -0,04 |

**Supplementary Table S7.** Correlation coefficients between the different alveograph parameters for doughs with ingredients for bread.

|  | P | L | G | W | P/L | Ie | Dmin | Dmax | SH |
| --- | --- | --- | --- | --- | --- | --- | --- | --- | --- |
| L | 0,00 |  |  |  |  |  |  |  |  |
| G | 0,01 | 1,00 |  |  |  |  |  |  |  |
| W | 0,78 | 0,51 | 0,51 |  |  |  |  |  |  |
| P/L | 0,59 | -0,79 | -0,79 | 0,07 |  |  |  |  |  |
| Ie | 0,56 | 0,33 | 0,33 | 0,87 | 0,11 |  |  |  |  |
| Dmin | -0,51 | 0,36 | 0,34 | 0,04 | -0,55 | 0,40 |  |  |  |
| Dmax | 0,99 | -0,01 | 0,00 | 0,79 | 0,60 | 0,61 | -0,44 |  |  |
| SH | 0,40 | 0,42 | 0,42 | 0,81 | -0,05 | 0,98 | 0,55 | 0,45 |  |
| K | 0,88 | -0,23 | -0,21 | 0,43 | 0,67 | 0,11 | -0,82 | 0,84 | -0,08 |

**Supplementary Table S8.** Overview of the conducted alveograph analyses, including type of flour, addition of other ingredients and changes in analysis conditions. The alveograph analyses were conducted according to the standard AACC method 54-30.02, apart from the exceptions noted. A total of 41 tests were carried out in triplicate, and parameters from the five pressure curves were applied in data analysis. Curves were removed if the inflation stopped prematurely.

| **Flour** | **Other ingredients** | **Analysis conditions** | **Number of curves** |
| --- | --- | --- | --- |
| Danish pastry flour (batch 4) | None |  | 15 |
| Danish pastry flour (batch 4) | Bread dough ingredients ^a^ | Ingredients cooled prior to analysis  Temperature of mixing bowl and resting chamber 18 °C and test chamber 15 °C  Mixing time of 10 minutes | 4 (11 out of 15 removed) |
| Danish pastry flour (batch 4) | Danish pastry dough ingredients ^b^ | Ingredients cooled prior to analysis  Temperature of mixing bowl and resting chamber 18 °C and test chamber 15 °C  Mixing time of 10 minutes | 3 (12 out of 15 removed) |
| Bread flour | None |  | 15 |
| Bread flour | Bread dough ingredients ^a^ | Ingredients cooled prior to analysis  Temperature of mixing bowl and resting chamber 18 °C and test chamber 15 °C  Mixing time of 10 minutes | 13 (2 out of 15 removed) |
| Bread flour | Danish pastry dough ingredients ^b^ | Ingredients cooled prior to analysis  Temperature of mixing bowl and resting chamber 18 °C and test chamber 15 °C  Mixing time of 10 minutes | 15 |
| Cake flour | None |  | 15 |
| Cake flour | Bread dough ingredients ^a^ | Ingredients cooled prior to analysis  Temperature of mixing bowl and resting chamber 18 °C and test chamber 15 °C  Mixing time of 10 minutes | 15 |
| Cake flour | Danish pastry dough ingredients ^b^ | Ingredients cooled prior to analysis  Temperature of mixing bowl and resting chamber 18 °C and test chamber 15 °C  Mixing time of 10 minutes | 14 (1 out of 15 removed) |
| Danish pastry flour (batch 1) | None | Water addition was adjusted according to water absorption | 15 |
| Danish pastry flour (batch 1) | 1 %, 2 %, 4 % or 8 % α-, β- or γ-cyclodextrin ^c^ | Water addition was adjusted according to water absorption | 180 |
| Danish pastry flour (batch 2) | None | Mixing time of 3, 4, 5, 6, 8, 10 and 14 minutes | 105 |
| Danish pastry flour (batch 2) | Danish pastry dough ingredients ^b^ | Ingredients cooled prior to analysis  Temperature of mixing bowl and resting chamber 18 °C and test chamber 15 °C  Mixing time of 4, 6, 8, 10, 12, 13 and 14 minutes | 61 (44 out of 105 removed) |
| Danish pastry flour (batch 3) | Industrial samples of Danish pastry dough | The dough is frozen and thawed at 5 °C before analysis  Temperature of mixing bowl and resting chamber 18 °C and test chamber 15 °C  Dough was not scraped down during mixing  Mixing time of 2, 4, 6, 8 and 10 minutes | 62 (13 out of 75 removed) |

^a^ 60 % demineralised water, 1 % sodium chloride and 0.8 % dry yeast (indicated in percentage of flour weight)

^b^ 50 % demineralised water, 6.7 % pasteurised eggs, 5.4 % sugar, 4.1 % compressed yeast, 3.5 % margarine, 1.5 % improver and 0.35 % sodium chloride (indicated in percentage of flour weight)

^c^ Indicated in percentage of flour weight
